# Developing and testing the usability, acceptability, and future implementation of the Whole Day Matters Tool and User Guide for primary care providers using think-aloud, near-live, and interview procedures

**DOI:** 10.1186/s12911-023-02147-x

**Published:** 2023-04-06

**Authors:** Tamara L. Morgan, Jensen Pletch, Emma Faught, Michelle S. Fortier, Mary Kate Gazendam, Kelly Howse, Rahul Jain, Kirstin N. Lane, Kaleigh Maclaren, Taylor McFadden, Jeanette C. Prorok, Zachary J. Weston, Jennifer R. Tomasone

**Affiliations:** 1 School of Kinesiology and Health Studies, Queen’s University, 28 Division Street, Kingston, ON Canada; 2 School of Medicine, Queen’s University, Kingston, ON Canada; 3 School of Human Kinetics, University of Ottawa, Ottawa, ON Canada; 4 Loyalist Family Health Team, Amherstview, ON Canada; 5 Temerty Faculty of Medicine, University of Toronto, Toronto, ON Canada; 6 Canadian Society for Exercise Physiology, Ottawa, ON Canada; 7 School of Exercise Science, Physical and Health Education, University of Victoria, Victoria, BC Canada; 8 Independent Communication Specialist, Ottawa, ON Canada; 9 Canadian Medical Association, Ottawa, ON Canada; 10 Canadian Frailty Network, Kingston, ON Canada

**Keywords:** Primary care, Prevention, Lifestyle medicine, 24-Hour Movement Guidelines, Qualitative research, Integrated knowledge translation, Think-aloud, Near-live

## Abstract

**Background:**

Canada’s 24-Hour Movement Guidelines for Adults have shifted the focus from considering movement behaviours (i.e., physical activity, sedentary behaviour, and sleep) separately to a 24-h paradigm, which considers how they are integrated. Accordingly, primary care providers (PCPs) have the opportunity to improve their practice to promote all movement behaviours cohesively. However, PCPs have faced barriers to discussing physical activity alone (e.g., time, competing priorities, inadequate training), leading to low frequency of physical activity discussions. Consequently, discussing three movement behaviours may seem challenging. Tools to facilitate primary care discussions about physical activity have been developed and used; however, few have undergone usability testing and none have integrated all movement behaviours. Following a synthesis of physical activity, sedentary behaviour, and sleep tools for PCPs, we developed the Whole Day Matters Tool and User Guide that incorporate all movement behaviours. The present study aimed to explore PCPs’ perceptions on the usability, acceptability, and future implementation of the Whole Day Matters Tool and User Guide to improve their relevancy among PCPs.

**Methods:**

Twenty-six PCPs were observed and audio–video recorded while using the Tool and User Guide in a think-aloud procedure, then in a near-live encounter with a mock service-user. A debriefing interview using a guide informed by Normalization Process Theory followed. Recordings were transcribed verbatim and analysed using content analysis and a critical friend to enhance rigour.

**Results:**

PCPs valued aspects of the Tool and User Guide including their structure, user-friendliness, visual appeal, and multi-behaviour focus and suggested modifications to improve usability and acceptability. Findings are further discussed in the context of Normalization Process Theory and previous literature.

**Conclusions:**

The Tool and User Guide were revised, including adding plain language, reordering and renaming sections, reducing text, and clarifying instructions. Results also informed the addition of a Preamble and a Handout for adults accessing care (i.e., patients/clients/service-users) to explain the evidence underpinning the 24-Hour Movement Guidelines for Adults and support a person-centered approach. These four resources (i.e., Tool, User Guide, Preamble, Handout) have since undergone a consensus building process to arrive at their final versions before being disseminated into primary care practice.

**Supplementary Information:**

The online version contains supplementary material available at 10.1186/s12911-023-02147-x.

## Introduction

Since October 2020, the Canadian 24-Hour Movement Guidelines for Adults (24HMG) have encouraged general population adults in Canada to make their “whole day matter” by striving for healthy levels of physical activity (PA), sedentary behaviour (SB), and sleep (i.e., movement behaviours) each day to achieve health benefits [1]. Regrettably, data from the Canadian Health Measures Survey supports that daily movement patterns of adults in Canada are poor; only 9% of adults met all 24HMG recommendations and 19% met none of the recommendations [2]. Adults who meet two or fewer of the recommendations have shown less favourable mental health outcomes [3] and cardiometabolic health indicators, such as waist circumference and serum glucose levels [2, 3]. With a new focus on the 24-h paradigm [1, 4], primary care providers (PCPs) have the opportunity to discuss movement behaviours an integrated manner to improve the health of adults in Canada. However, this opportunity comes with unique challenges for PCPs’ decision-making.

While PA counseling and prescription have been growing trends among PCPs, research suggests the frequency of PA discussions in primary care is low [5, 6]. One study among 10 Québec family medicine groups reported that, in a sample of adults with and without chronic illnesses, 52% had their PA assessed and 21% were counseled about PA by a family physician or nurse in 2019 [7]. Further, a survey among 1751 US physicians and nurse practitioners identified that while 92.7% of PCPs encouraged adults at-risk for cardiovascular disease to engage in more PA, only 25.6% wrote a PA prescription and only 15.1% referred to a behavioural counsellor for follow-up [8]. Discussions with PCPs on improving SB and sleep for health promotion occur even less frequently [9, 10]. Collectively, PCPs have reported low PA, SB, and sleep health promotion knowledge, and low confidence, skill, and frequency in discussing movement behaviours [7, 9–13]. Given these barriers, addressing three movement behaviours may seem challenging.

Systematic reviews [11, 14] and recent studies [9, 15] have suggested that PCPs who are equipped with materials, strategies, or tools to direct them through movement behaviour discussions are more likely to promote these behaviours to adults accessing care. Recently, we conducted a scoping review to evaluate tools developed for guiding discussions on PA, SB, and/or sleep between adults and PCPs in Canada and analogous countries [16]. Of the 61 tools we discovered, an overwhelming 51 tools catered solely to PA discussions. Comparatively, we found only one tool for sleep health promotion (i.e., rather than for sleep disorders or diagnoses), seven tools combining PA and SB, and two tools combining PA and sleep [16]. Evidently, tools to guide integrated discussions on 24HMG behaviours are currently unavailable. Thus, the development of a 24HMG discussion tool may help PCPs overcome the challenge of discussing all three movement behaviours.

**Fig. 1:**
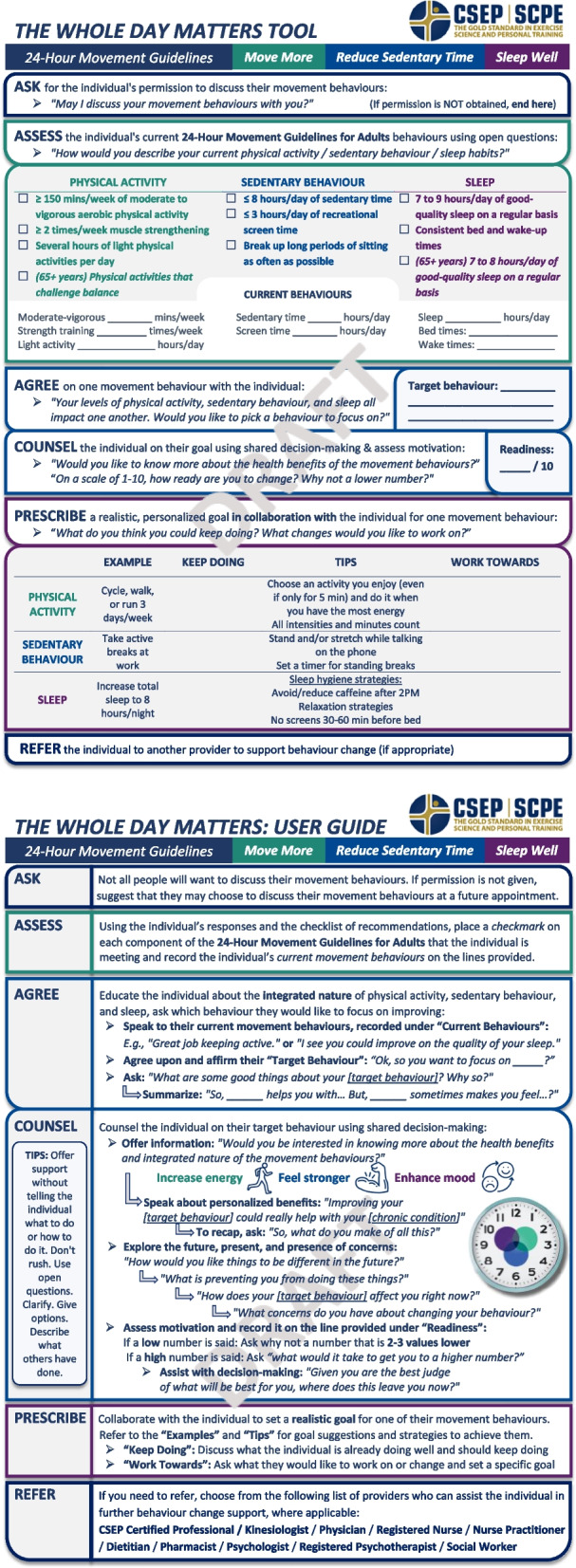
The Whole Day Matters Tool and User Guide pre think-aloud

However, implementing such a tool in primary care presents another challenge given the suboptimal use of research evidence to fill gaps in practice [17]. Theories, models and frameworks have informed efforts to close knowledge-practice gaps and guide implementation [18]. The Knowledge to Action Framework explains how knowledge may be used in practice [19] and was the guiding framework for this study. Our work is part of the larger knowledge translation (KT) process for the 24HMG [20], whereby the 24HMG development process comprised the “knowledge funnel” of the Knowledge to Action Framework, resulting in the guidelines as the knowledge product. Then, we embarked on the “action cycle” with our scoping review, which identified a gap in tools for 24HMG discussion in primary care. To bridge this gap, we applied our scoping review findings to develop a 24HMG discussion tool and accompanying manual for PCPs—coined as “The Whole Day Matters Tool and User Guide” (Fig. 1). However, before closing this gap and disseminating and implementing the Tool and User Guide, their usability and acceptability to PCPs must be determined [19].

Think-aloud and near-live methods have been used in health care research to solicit feedback on the usability of decision-making tools [21, 22]. In the think-aloud method, participants verbalize their thoughts while performing a task, whereas in the near-live method, they engage with an actor while performing a task [21]. To our knowledge, think-aloud and near-live methods have only been used non-consecutively, such as in a two-phased research study, rather than in sequence (e.g., [21, 22]). The use of these two methods in sequence may help access users’ initial thoughts on usability and their thoughts after gaining familiarity with the Tool and User Guide, which may have methodological implications for tool developers. *Usability* is defined as the degree to which an innovation may be used efficiently, effectively, and satisfactorily to achieve specific goals in a specific context [23]. Moreover, and clarifying varying terminology used in implementation research, Proctor and colleagues [24] define *acceptability* as the extent to which an innovation and its components are agreeable to its users. However, exogenous factors may also hinder or promote the normalization of innovations into practice. Normalization Process Theory (NPT) explains how material practices, such as decision-making tools, are integrated within social settings from an action standpoint [25, 26]. NPT helps understand implementation determinants regarding what actions people take to embed a given practice within their setting [25], which may inform tool revisions that better support future implementation success.

In the present study, we tested the usability of the Tool and User Guide via consecutive think-aloud and near live procedures and explored acceptability and implementation determinants via follow-up interview questions. We operationalized usability as the extent to which PCPs felt they could use the Tool and User Guide to efficiently, effectively, and satisfactorily discuss movement behaviours in a primary care appointment, and acceptability as the extent to which PCPs’ (dis)liked and (mis)understood the Tool and User Guide [23, 24]. While the findings of usability and acceptability testing can be used to improve tools, few studies have led to revised versions of tools and their implementation [27]. Therefore, the purpose of this study was to explore PCPs’ perceptions of the usability, acceptability, and future implementation of the Whole Day Matters Tool and User Guide to make revisions to enhance their relevancy to PCPs. Specific research questions were (i) What are PCPs’ perspectives on the usability and acceptability of the Tool and User Guide? (ii) To what extent do PCPs use the Tool and User Guide and why? and (iii) What considerations do PCPs have for implementing the Tool and User Guide in future practice?

## Methods

### Tool and User Guide Mock-up

This research was carried out using an integrated knowledge translation (iKT) approach, which is a model of research that involves ongoing collaboration with individuals who are positioned to recognize research-practice gaps and help implement evidence to close said gaps [28, 29]. Applying iKT, we formed a working group of experts in the fields of medicine, exercise psychology, health promotion, communications, and KT [28, 29]. Applying our scoping review findings [16], our working group conceptualized and developed a mock-up of the Whole Day Matters Tool and User Guide over two 90-min meetings and email communication (September–November 2021). First, the Tool and User Guide were based off of a modified version [30] of the 5 A’s Framework, which was developed as a brief PA counselling strategy for primary care [31]. Using the modified 5 A’s [30], the following stages are covered in the Tool and User Guide (*original 5 A’s Framework stages listed in italics* [31]): Assess (*Ask*), Advise a target behaviour (*Advise*), Counsel (*Assess [readiness]*), Prescribe (*Assist*), and Refer (*Arrange*). Additionally, the Tool and User Guide were designed to: (i) be quick to use and limited to one page; (ii) be usable in PDF format or printed; (iii) follow the 24HMG branding and include graphics and limited yet informative text; (iv) use generic statements that could apply to adults with or without chronic conditions, given that PCPs do not always exclusively serve either/or; (v) use principles of motivational interviewing including having a person-centered approach, using open-ended questions, using a scale to assess readiness, build motivation for change, and discuss barriers and motivators to change [32]; and (vi) be accompanied by a user guide including additional instructions (e.g., steps, definitions).

### Usability and acceptability testing

This study followed an observational design underpinned by critical realism, which draws from a realist ontology and interpretivist (or constructivist) epistemology [33]. Realist ontologies assert that reality exists apart from ourselves, but may never be truly or completely understood [34] while interpretivist epistemologies assume that participants’ past experiences are subject to researchers’ interpretation [33]. In an interpretivist lens, acknowledging researchers’ positionalities is important as their experiences also influence the research process [34]. The first author is a white, cis-gender, young adult female with two years of post-secondary training in a design program as well as a bachelor’s and master’s in human kinetics and exercise psychology who, at the time of the study, was a third year doctoral candidate in health promotion. In the present study, participants’ diverse backgrounds as various PCPs (e.g., physicians, nurses, dietitians) influenced their perceptions of the Tool and User Guide and these perceptions were verbalized through interactions with, and interpreted by, the first author [33, 35]. However, to ensure all perspectives were heard and no voices were overemphasized, the first author was mindful of participants’ diverse experiences.

Reporting of this study is in line with the Standards of Reporting Qualitative Research [36] (Additional file 4: S-Table 1). Ethics clearance from the Queen’s University General Research Ethics Board (TRAQ#: 6034390) was given prior to commencing recruitment.

#### Participants and procedure

This study recruited PCPs, who are the target users of the Tool and User Guide. Eligible PCPs included those regularly involved in health promotion/health behaviour discussions with adults accessing care in a primary care setting in Canada [10, 37–41], including: physicians, residents, nurses, nurse practitioners, dietitians, pharmacists, social workers, and psychologists, and registered psychotherapists working in a family health team in Ontario, Canada. Our target sample size was 25 participants (i.e., five per each medicine, nursing, diet/nutrition, pharmacy, and psychology fields). Research supports that a sample of five participants is adequate to capture the large majority of usability problems in a think-aloud study [42]. As the Tool and User Guide were developed in English, participants had to be able to read and speak in English.

The recruitment strategy was co-developed with two local family health teams and the wellness centre at the university this research was conducted at, where the research team leveraged existing professional networks. Three contacts (one at each family health team and one at the wellness centre) sent an email in November 2021 to invite eligible staff to participate by clicking a link to a preliminary survey and registration form in the online survey platform Qualtrics. Participants indicated their consent for audio and video recording and use of anonymized quotations by clicking ‘next’ on the first survey page. Questions on demographics, PCPs’ profession, and knowledge and awareness of the 24HMG were included in survey. Eligible participants were contacted to arrange a meeting over Microsoft Teams, which was expected to last approximately 35 min. Meetings were recorded using the software Camtasia, which allowed us to document all screen activity [43]. Compensation in the form of an e-gift card worth $75 CAD was sent to each participant within one week of their participation.

#### Think-aloud and near-live

Think-aloud and near-live methodologies were used to gather participants’ perspectives on the usability of the Tool and User Guide. The concurrent think-aloud method showcases the cognitive thought processes that arise while one is simultaneously executing a particular task [44], which are thought to be a valid representation of one’s working memory [45] and help ascertain superficial usability issues [22]. Working memory is relevant to tool usability as it concerns information processing for decision-making and reasoning [46]. Compared to retrospective think-aloud, concurrent think-aloud methods have been shown to detect a significantly greater number and severity of usability problems in less time [45]. Alternatively, the near-live method explores deep usability issues in a more realistic setting, which may help discover fundamental issues with workflow [21, 22]. Prior work has shown that think-aloud and near-live procedures both access the majority of usability outcomes (i.e., content, understandability, visibility, navigation, and workflow), but that the near-live method captures more information about usability and usefulness for real-life practice [22]. We applied the two methods sequentially to capture both superficial and deep usability problems as well as insight from participants’ increasing familiarity with the Tool and User Guide, given they are asked to comment on usability twice, once in their first-ever use (i.e., think-aloud), and once after becoming acquainted with the Tool and User Guide (i.e., near-live).

Online meetings were facilitated by the first author and began with a statement of the study purposes and a brief warm-up task unrelated to the Tool and User Guide to orient participants to the think-aloud procedure by having them practice verbalizing their thoughts [44, 47]. Feedback was given to participants when needed to ensure participants comprehended the essence of the think-aloud. The think-aloud task involved the facilitator screen-sharing a copy of the Tool and User Guide in PDF format, providing participants mouse control, and reading a scenario (Additional file 1) that asked participants to attempt to use the Tool and User Guide to discuss movement behaviours in a hypothetical clinic appointment. The think-aloud was expected to take approximately 5–10 min.

The near-live task immediately followed the think-aloud and involved the facilitator screen-sharing the Tool and User Guide again, providing mouse control, then reading a different scenario, which asked participants to use the Tool and User Guide to guide a conversation with a mock service-user. A brief description of the mock service-user, who joined the Teams meeting solely for the near-live task, was given explaining their history and purpose of visit. The mock service-user (second author) was an undergraduate student intern who had completed a course in motivational interviewing and was trained in the role over two two-hour sessions. During these training sessions, the third author (a medical student who was acquainted with the Tool and User Guide mock-up) played the role of a PCP using the Tool. The first author (a doctoral student in health promotion) gave feedback on the mock service-user’s consistency acting in accordance with their established profile and on the quality and plausibility of their responses to discussions arising from the third author’s use of the Tool. Six scenarios were used to ensure that participants’ perspectives on the usability of the Tool and User Guide to guide discussions on each PA, SB, and sleep were represented, with permutations per someone who is either motivated or unmotivated for behaviour change. The scenarios were co-created with the medical student on the working group, reviewed by our physician contact at each family health team, and revised per their feedback. The near-live was expected to take approximately 5–10 min. Detailed field notes were taken by the facilitator during both the think-aloud and near-live tasks.

#### Interviews

Meetings were concluded with a semi-structured debriefing interview to explore participants’ reflections on the usability, and perceptions of acceptability and future implementation, of the Tool and User Guide’s in clinical practice. The debriefing interview was informed by an interview guide and the first author’s field notes to probe participants’ think-aloud and near-live encounters. Additional questions informed by NPT asked how uptake and use of the Tool and User Guide could be supported or inhibited in clinical practice [26], which was done to proactively investigate implementation barriers and facilitators. Interview questions were developed to speak to coherence (i.e., participants’ conceptualizations about the Tool and User Guide), cognitive participation (i.e., participants’ commitment to the Tool and User Guide), and collective action (i.e., factors that may promote or constrain use of the Tool and User Guide in practice). Reflexive monitoring was omitted as it concerns how a practice is appraised, which would come during or after the Tool and User Guide are embedded in practice [25]. Interviews were concluded by asking if participants had any remaining questions or comments and stating that a follow-up email would be sent about compensation. The think-aloud and near-live protocols, mock service-user profiles, and interview guide are shown in Additional files 1, 2 and 3.

#### Analyses

Recordings were transcribed verbatim by the second author, anonymized, and imported into NVivo version 12 [48] for analysis. Content analysis was performed to broadly describe participants’ perceptions of the Tool and User Guide and offer an action plan for revisions [49]; this was achieved through creating categories that could speak to components (i) worth keeping or (ii) in need of amendment. Two additional categories were created that spoke to (i) barriers and facilitators and (ii) the work PCPs do to support embedding the Tool and User Guide in practice, which were made possible through the use of NPT-informed questions in the interview guide. Research has labelled content analysis as either manifest, examining surface meaning, or latent, examining underlying meaning [50]. Manifest content analysis was performed by the first author in two stages (creating initial codes and categorization/sub-categorization) and the second author acted as a critical friend to relay questions or agreement with the appropriateness of the codes, categories, and sub-categories after each stage [49]. Categories and sub-categories were further refined following discussion with the last author, who acted as a second critical friend.

## Results

Thirty-two eligible PCPs expressed an interest in participating. After being invited to partake, one participant withdrew due to family obligations, one withdrew due to difficulty joining the Teams meeting, three did not respond to the invitation, and one was scheduled to participate but did not attend and was unable to reschedule. From November to December 2021, 26 PCPs consented to participate and completed the preliminary survey and the think-aloud, near-live, and interview procedures. Meetings lasted an average of 45.57 (SD = 10.29) minutes [think-aloud *M* = 8.39 (SD = 3.48) minutes; near-live, *M* = 11.58 (SD = 4.31) minutes; interview *M* = 17.58 (SD = 4.90) minutes]. Most participants were family medicine residents (57.7%), identified as female (73.1%), had been practicing for an average of 4.49 (SD = 7.03) years, and were familiar with the 24HMG (65.4%); however, only 19.2% had sufficient knowledge of the 24HMG to correctly state all three of its components (i.e., PA, SB, and sleep). Half of participants (50%) identified as white, non-mixed descent. All participants were employed in Ontario, Canada at the time of the study. All participant characteristics are shown in Table 1.

**Table 1:** Participant demographic and occupational characteristics (*N* = 26)

	***M*** ** (SD)**
**Years in Practice**	4.49 (7.03)
	***n*** ** (%)**
**Gender Identity**	
Woman	19 (73.1%)
Man	7 (26.9%)
**Self-identification of Descent**	
White	13 (50.0%)
Chinese	7 (27.0%)
Other^a^	6 (23.0%)
**Profession**	
Family medicine resident	15 (57.7%)
Family medicine physician	4 (15.4%)
Nurse	3 (11.5%)
Dietitian	2 (7.7%)
Social Worker	2 (7.7%)
**Community Served**	
Urban	17 (65.4%)
Suburban	9 (34.6%)
**Population Served**^**b**^	
Adults 18–64 years	26 (100%)
Adults 65 + years	24 (92.3%)
Adults with diabetes	23 (88.5%)
Adults who are pregnant	23 (88.5%)
Adults with osteoporosis	20 (76.9%)
Adults with cancer	19 (73.1%)
Adults with Alzheimer’s disease	17 (65.4%)
Adults with Parkinson’s disease	16 (61.5%)
Adults with multiple sclerosis	12 (46.2%)
Adults with spinal cord injury	10 (38.5%)
Adults with mental health concerns	2 (7.7%)
Pediatric populations	1 (3.8%)
**Familiarity with the 24-Hour Movement Guidelines**	
Not familiar at all	9 (34.6%)
Familiar	17 (65.4%)
*Just heard the name*	*11 (42.3%)*
*Somewhat familiar*	*6 (23.1%)*
**Knowledge of the 24-Hour Movement Guidelines (open text)**	
Correctly identified the three main components (i.e., physical activity, sedentary behaviour, and sleep)	5 (19.2%)
Correctly identified two of the three main components	4 (15.4%)
Correctly identified one of the three main components	3 (11.5%)
Did not know/incorrect response/no response	14 (53.9%)
**Knowledge of the 24-Hour Movement Guidelines (multi-select)**^**b**^	
At least 150 min of moderate to vigorous physical activity a week, including at least 2 days of muscle strengthening activities per week	13 (50.0%)
Several hours of light physical activity, including standing	4 (15.4%)
Limit sedentary time to 8 h or less per day	8 (30.8%)
7–9 h of good quality sleep on a regular basis, with consistent bed and wake-up times (for adults 18–64 years)	12 (46.2%)
7–8 h of good quality sleep on a regular basis, with consistent bed and wake-up times (for adults 65 + years)	7 (26.9%)
Perform physical activities that challenge balance	5 (19.2%)
Replacing sedentary behaviour with additional physical activity and trading light physical activity for more moderate to vigorous physical activity, while preserving sufficient sleep, can provide greater health benefits	7 (26.9%)
Did not know/unsure	3 (11.5%)
Incorrect response	3 (11.5%)

*M(SD)* mean (standard deviation)

^a^Other descents self-identified included Black, Arab, Korean, South Asian, Southeast Asian, Indian, Caribbean, and mixed descent

^b^Category total is greater than sample size due to option to select multiple responses

In the next section, results are presented across four categories: 1) positive perceptions, 2) usability challenges, 3) barriers and facilitators to future embedding in practice, and 4) processes of using the Tool and User Guide. Illustrative quotations are provided throughout each category.

### Positive perceptions of the Tool and User Guide

A number of PCPs voiced their approval for one or more aspects of the Tool during the think-aloud and near-live. Many found that the Tool was concise yet contained a helpful amount of detail and therefore considered using it in their practice. A large number of PCPs appreciated the combined focus on movement behaviours.

Further, PCPs appreciated how the Tool supported their discussion, claiming it was user-friendly, person-centered, and provided a helpful structure to frame the conversation. PCPs liked how the 24HMG recommendations were broken down in ASSESS, how AGREE asked them to collaborate on choosing a target behaviour, the reminder to set a goal, and the scaling question to assess readiness in COUNSEL. One resident summarized:

*“[The Tool] clearly shows… the steps whenever you are working on behaviour modification with a person… it is just really easy when you are talking to someone to forget that they are the one that has to do the change so… you should really base it on what they are wanting to do.” [Jayden, interview]*

Some PCPs also highlighted how the Tool and User Guide could serve as an educational resource and inform PCPs and adults accessing care of the new 24HMG. Another resident expressed:

*“We haven’t been taught how to take a good physical activity and sedentary behaviour history. I thought it was kind of helpful to have a list of how you can go about asking questions on those behaviours, and having the guidelines… that was a helpful add that I had not seen before.” [Giselle, interview]*

Positive perceptions on the Tool’s visual appeal were also expressed, pertaining to the organization via columns, rows, and headings, and the use of the 24HMG colours to distinguish PA, SB, and sleep. One resident said:

*“I really like the colours… It is nice to structure and have different words, colours, headings, it is very appealing and easy to read in a pinch.” [Philippe, interview]*

Finally, some PCPs reported that the User Guide was convenient in explaining how to use the Tool and locating more information to support their discussion when needed, and could be used side-by-side with the Tool, like a booklet.

However, two PCPs indicated that, while they referred to the User Guide, they may not need to use it each time. One resident said:

*“I think over time I would just need the first page just to kind of write notes and things like that.” [Faisal, interview]*

Overall, PCPs appreciated the content, structure, and look of the Tool and User Guide, as well as how they functioned as side-by-side resources, with some PCPs feeling that the Tool and User Guide could be used to educate about 24HMG promotion.

### Challenges to using the Tool and User Guide

Uncertainty was also voiced by PCPs when using the Tool and User Guide during the think-aloud and near-live. Some PCPs were unclear on whether the term “movement behaviours” encompassed just PA, or also included SB and sleep. A few PCPs considered PA and SB as occupying opposite ends of the same spectrum, rather than as the two co-dependent constructs they are.

Alternatively, other PCPs including a dietitian recognized that “movement behaviours” meant all three behaviours:

*“I just want to say physical activity but I know movement behaviours is more than that.” [Clara, think-aloud]*

Relatedly, PCPs were uncertain about specific areas in the Tool, including not knowing how to use the checkboxes or 24HMG benchmarks in ASSESS, how to interpret answers on the readiness scale in COUNSEL, or how to fill out the table in PRESCRIBE. Some also had difficulty navigating through the second half of the Tool, from AGREE to COUNSEL to PRESCRIBE, and difficulty with REFER, such as this nurse:

*“I don’t know who all to refer to, so if like I wanted to refer someone for more physical activity, like sleep is a bit easier because there’s sleep clinics, umm I don’t know who to refer to, so the refer was a bit challenging for me.” [Jamie, interview]*

Some PCPs felt that aspects of the Tool and User Guide’s appearance deterred its use. Predominantly, PCPs commented that the Tool and User Guide had too much text and not enough white space (i.e., for visual appeal or to have room to document in the Tool), and was not person-centered as a result. One resident mentioned:

*“Looking at the tool it seemed more geared towards the provider that was asking the patient so… that didn’t seem like the most patient friendly sheet to take with them.” [Levi, interview]*

Finally, PCPs noted some challenges with the User Guide, namely, that it was awkward to refer to it during a conversation or unclear on how to use it overall. Perhaps related, three PCPs reported missing the counseling prompts in the User Guide. One social worker explained:

*“The only thing was like the counsel because I didn’t have a lot of time to look over [the User Guide] that when I scroll down to [the User Guide], that counsel kind of offers different tips and different things able to bring up in terms of conversation. So it is not just what is in that first little box [in the Tool], that there is more to it and you wouldn’t know if there was not that second page.” [Nora, interview]*

In general, PCPs perceived the counseling prompts in the User Guide as helpful but too lengthy. In the Tool, PCPs felt challenged by the 24HMG terminology, aspects of flow, and overabundance of text.

### Barriers and facilitators to future embedding in practice

Multiple factors that PCPs saw as hindering or promoting acceptability and future implementation of the Tool and User Guide were identified. PCPs thought that many adults accessing care would not easily understand the terms “physical activity intensity”, “sedentary behaviour”, and “movement behaviours” and would not be able to accurately quantify their movement behaviours. Thus, PCPs were deterred from such terminology with the mock service-user.

Conversely, some PCPs indicated having relevant background knowledge about concepts in the Tool (i.e., movement behaviour promotion, motivational interviewing, the 5 A’s framework) that helped their conversation flow more naturally in the near-live scenario. One resident highlighted:

*“Knowing the guidelines is probably ideal… and being able to do goal-setting not just what was in the tool but even just having basic SMART goals or goals with objectives… and then* o*bviously the counseling and maybe some motivational interviewing techniques that we learn through residency to be able to assess their readiness for change.” [Mhairi, interview]*

Barriers in the practice setting were also mentioned, including time constraints, competing priorities in appointments, and high prevalence of poor movement behaviours and motivation among adults accessing care. Some PCPs expressed friction between the topic of movement behaviours being undervalued in medicine and the challenge of not making lifestyle discussions feel judgemental to the adult accessing care. One resident exemplified this well:

*“I think a lot of people go to the doctors and feel very attacked and feel like ‘I am doing all these things already but my doctor keeps telling me to do more things’ and then its frustration.” [Grant, think-aloud]*

Additional facilitators encompassed how the Tool and User Guide could be modified to improve efficiency, understandability, and flow, supplemented with additional resources to improve uptake, and disseminated to PCPs. First, to rectify challenges using the Tool, PCPs recommended modifying the prompts within each Tool section (e.g., ASK) and the instructions in the Tool and User Guide to simplify terminology. One dietitian said:

*“I mean it’s nice to have sample question and things, but it is very unlikely that I would read everything off the sheet the way that it is written.” [Clara, interview]*

Moreover, PCPs requested clarification on whether to focus on one or multiple movement behaviours and a change in wording from asking ‘*whether* to pick a behaviour’ to ‘*which* behaviour to pick’ in AGREE. For example, one resident thought:

*“I think that it would be a good idea to target a specific area to start with and then in future clinical visits we could talk about the other areas. But at the same time there might be patients that want to think about all the areas which I think would be fine too.” [Mia, think-aloud]*

Lastly, PCPs wanted additional points on 24HMG tips and benefits in COUNSEL, more example activities in PRESCRIBE, and more guidance in REFER beyond what was listed in the User Guide (e.g., availability of accessible services or providers, when and why to refer, and whether it is optional). Regarding the latter, one resident stated that:

*“Referring is great but often these things are not covered and not available, so great in a high resource situation but not in this situation, in many places in Canada, and many people’s selective insurance.” [Raniyah, think-aloud]*

A number of PCPs articulated that they would reorder sections within the Tool. For instance, many PCPs preferred goal-setting to come after evaluating movement behaviours in ASSESS and before gauging readiness for change in COUNSEL.

Some PCPs stated the importance of becoming familiar with and personalizing the Tool and User Guide before first using them. In fact, many reported increased familiarity and comfort with the Tool during the near-live task compared to their encounter with the Tool during the think-aloud, such as this nurse:

*“… it felt a little bit awkward on the think-aloud but then I sort of got my head around the tool a little bit better so it was easier to use for counseling after that.” [Clara, interview]*

The development of additional resources for PCPs and adults accessing care was also highlighted as a potential facilitator. Participants advocated for including a preamble to explain the Tool and User Guide, background information, evidence, and statistics about the 24HMG, training (e.g., workshop, webinar), and more examples of how to meet the 24HMG as discussion prompts.

Furthermore, numerous PCPs desired more public-facing materials to support using the Tool and User Guide, such as a handout with more images, less text, education on the 24HMG in lay terms, areas where adults accessing care could write out their goals, and tips for achieving behaviour change. One physician relayed:

*“I would love a handout on easy physical activity changes or easy sedentary behaviour changes or easy sleep changes that are consistent with the guidelines that I can send with people to take home. There is a lot of research that says the conversations we have with patients in clinic, they don’t retain it, which isn’t surprising, no one can retain everything and so I would talk to them about things like reducing caffeine and reducing screen time but having a handout would be really helpful if I could email or print out for them.” [Brienne, think-aloud]*

Many PCPs expressed that the Tool and User Guide aligned well with their professional roles (e.g., performing documentation, counseling, writing prescriptions) and scope of practice (e.g., diabetes education, obesity counseling, mental health checks, disease prevention).

Conversely, other PCPs felt the Tool and User Guide did not align with their professional role. For instance, one dietitian felt that sleep was not their area of expertise and the two social workers were unsure of their role in using the Tool.

However, both social workers admitted that promoting movement behaviours to improve mental and physical health could be relevant to social work, suggesting they may want to use the Tool and User Guide in the future. This social worker claimed:

*“This is a holistic approach that needs to find balance between these three things… that sleep and rest are just as important and if we don’t have that balance then that is sort of throwing your body off.” [Kendall, interview]*

Dissemination of the Tool and User Guide was recommended to signal PCPs to use the Tool and adults accessing care to initiate conversations about the 24HMG. Suggestions included providing a link to the 24HMG website and using appropriate dissemination channels to reach PCPs, such as displaying the Tool in waiting rooms, posting it on walls in clinics, or providing PCPs print-outs of the Tool. This physician stated:

*“… something that can be passed around clinics or posters we can put up in waiting rooms, for example, to prompt discussion… so if there was a ‘talk to your doctor’ about the 24-Hour Movement [Guidelines] I think that would help make our lives a lot easier.” [Catherine, interview]*

Many PCPs expressed that having the Tool and User Guide integrated within the electronic medical record (EMR) would promote use. However, several PCPs also indicated that paper copies, either visible on a desk or readily available in a drawer, might also work. Most of all, it was noted that the Tool and User Guide should each be kept at their one-page length.

### Processes of using the Tool and User Guide

Lastly, PCPs took different approaches to executing each Tool section (e.g., ASK, ASSESS), used the Tool in a collaborative manner, and tailored the Tool to their preferences. Most PCPs emphasized the benefit of explaining the connection between the 24HMG and the specific health condition(s) of the adult accessing care in ASK and AGREE, to promote buy-in. One resident expressed that the details in the User Guide were helpful to this end:

*“That is great, yeah like here, improving your target behaviour with your chronic condition so putting it in the context of their past medical history and limitations.” [Grant, think-aloud]*

Additionally, a number of PCPs completed ASSESS using quantitative questions (e.g., *“How many hours would you say you spend sitting on an average day?” [Harvey, near-live]*); however, the majority of PCPs preferred to assess movement behaviours open-endedly at first (e.g., “*How would you describe your current level of physical activity?*” *[Sofie, near-live]*), then quantify against the 24HMG benchmarks. Most PCPs assessed SB via questions about the mock service-user’s work environment. For instance, this resident asked:

*“How would you find your activity level is like kind of at work or during the bulk of your day?” [Ashton, near-live]*

In COUNSEL, some PCPs claimed they would not use the quantitative scale to evaluate readiness for behaviour change, but would either intuit readiness or ask in an open-ended manner. Nevertheless, many PCPs appreciated the scaling questions, with some preferring to ask “why not a *lower* number” and others preferring to ask “why not a *higher* number” to solicit the reasoning behind the chosen number. In PRESCRIBE, PCPs largely focused on improving one movement behaviour and ensuring the agreed behaviour change was attainable. Numerous PCPs highlighted the need to discuss accessible options for movement behaviours. One social worker voiced:

*“… make it feel for people like that they have options within their homes and I think a big issue for my clients is that they overcommit… and I think that’s when I see some of that like low follow-through that actually isn’t sustainable.” [Kendall, interview]*

In REFER, PCPs’ approaches included following up on the same or different movement behaviours and recommending community resources or social support to adhere to behaviour change. This social worker articulated:

*“Sometimes in family health teams they have a walking group, or if there are other types [of programs] in the community that would be helpful.” [Nora, interview]*

How PCPs used the Tool collaboratively included soliciting the mock service-user’s opinion and preferences in the behaviour change plan when initiating the conversation (i.e., asking whether they want to discuss movement behaviours), agreeing on a target behaviour, and setting a goal. For PCPs, this collaboration was meaningful as it upheld their values of person-centeredness, shared decision-making, and respect for the adult accessing care. One resident expressed:

*“... to have the patient say ‘this goal works more’ and have them follow along and be engaged. Umm, that’s the piece that I find the most important in the tool.” [August, interview]*

Further, reaching an agreement was seen as a means to ensure the behaviour change plan was relevant and realistic to the adult and was able to be monitored to track progress.

Lastly, PCPs stated that they would customize their approach to embedding the Tool and User Guide in their practice in two main ways. First, PCPs stated they would want to use the Tool over time, including merely mentioning the potential to use the Tool at a future appointment to ‘plant the seed’. Second, PCPs explained that they would use some or all sections of the Tool depending on the length or reason for the appointment, including wanting to use the Tool in an entirely separate appointment, like a wellness check. This resident claimed:

*“I think that with either sleep, sedentary behaviour, or physical activity there is no wrong option to lead down. I mean even If I got a little forward with one section in one visit, this is all stuff that I would be seeing a person for a few times at least.” [Ashton, interview]*

In sum, PCPs used the Tool and User Guide in varying ways, followed a collaborative, person-centered approach, and stated they would incorporate the Tool and User Guide to different degrees in their practice.

## Discussion

Integrated discussions on movement behaviours have not been common practice among PCPs prior to or following the launch of the 24HMG for Adults in October 2020. To address this gap, we developed the Whole Day Matters Tool and User Guide and explored their usability to improve their relevancy to PCPs. In the present study, 26 PCPs offered their perceptions of the Tool and User Guide’s usability, acceptability, and future implementation and suggested improvements to their visual appeal, ordering, and clarity.

### Positive perceptions of the Tool and User Guide

Positive perceptions pertained to the Tool’s content, user-friendliness, organized structure, colours, and potential as an educational resource. From the perspective of NPT, considering an innovation as important and useful is central to internalizing it as something valuable to one’s work [25, 51]. Among PCPs, valuing the Tool and its components speaks to a sense of coherence with the Tool [51]. Conversely, both a presence and lack of coherence was observed with the User Guide as it was seen by some as useful in providing additional explanations on the Tool but by others as unnecessary. The User Guide was intended as a document to orient PCPs to the Tool and/or to be referred back to sporadically as a ‘refresher’; thus, it is understandable that PCPs may use it to varying degrees. Interestingly, two PCPs who indicated the User Guide was unnecessary were physicians, which may point to the value of the User Guide as a training document rather than a frequently used resource. A Cochrane review of 215 studies indicated a moderate certainty of evidence that educational interventions slightly improved PCPs’ compliance with a desired practice compared to no intervention [52]. Perhaps, the User Guide could function as brief education to ensure PCPs’ compliance with the Tool over time. Future research is needed to evaluate whether the User Guide as a standalone educational tool or as a resource paired with a training session (e.g., online module, in-person event) would be better suited as an intervention to improve use of the Tool.

### Challenges to using the Tool and User Guide

Challenges pertained to unfamiliar terminology, visual appeal (i.e., too much text), and confusion about how to use certain components in the Tool. Similarly, the User Guide was perceived as awkward to reference during the near-live encounter and thus unlikely to be used during a real appointment. In light of NPT, PCPs’ challenges reduced the likelihood that they would use the Tool and User Guide in practice, representing a lack of internalization and coherence [51]. PCPs held varying knowledge of 24HMG concepts and mixed comprehension of what the Tool and User Guide required of them, known in NPT as individual specification [51]. Only some PCPs recognized that the Tool was meant for discussing the integrated nature of movement behaviours, potentially due to a lack of knowledge about the 24HMG. Other decision aid research has attributed differences in coherence to a lack of commitment, which could be amended by using a clinical champion [53]. Research has indicated that decision aid implementation has targeted human and technological factors over organizational factors (e.g., leadership, champions) [54] and that champions can increase implementation effectiveness [55]. Therefore, it appears worthwhile for tool developers to mobilize organizational factors, including champions, across clinics to support tool embedment. Using an iKT approach to tool development may be one way to initiate relationships with potential champions, by involving PCPs who may later use the Tool in all research stages [28, 29]. Previous tool development research in primary care engaged a clinical champion using an iKT approach and found that it strengthened the applicability of their tool and promoted its implementation [56, 57]. As the Tool and User Guide were developed for use by multiple types of PCPs (e.g., physicians, nurses, dietitians), several champions may be needed to promote uptake and use among all PCP groups.

### Barriers and facilitators to future embedding in practice

Factors that could facilitate or inhibit normalization of the Tool and User Guide in primary care were noted. Primarily, PCPs reported that adults accessing care may not understand movement behaviour terms. Adults’ insufficient knowledge about the 24HMG and suspected incoherence with the Tool and User Guide [51] could be due to the current shift in terminology toward “movement behaviours”, which is unfamiliar territory for most of the general public. In support, a recent systematic review conveyed that general population adults found that technical terminology, such as “movement”, “sedentary”, and “vigorous [intensity]”, needed better description within public health PA and SB guidelines [58]. Further, some PCPs reported dissonance between their professional role and discussing movement behaviours, whereas others reported coherence, which may hinder or enable acceptance of the Tool and User Guide, respectively [51]. Multiple initiatives could guide this culture shift toward discussing movement behaviours. For instance, workshops could be offered to educate PCPs on movement behaviour terminology with a motivational interviewing component to support a non-judgemental, person-focused approach [32] and 24HMG content could be embedded in the medical curriculum (c.f., [59]).

Additionally, PCPs considered movement behaviours an underutilized topic in primary care but confessed that, when these discussions do occur, adults may feel judged about the amounts of PA, SB, and/or sleep they engage in. From the coherence lens of NPT, this paradox exemplifies PCPs’ differentiation between the Tool and their current practice, both unfavourably and favourably [51]. Unfavourably, conversations guided by the Tool and User Guide may feel more pejorative than typical primary care conversations, where movement behaviours are not habitually discussed. Favourably, 24HMG discussions could promote an underrated topic, which could normalize and destigmatize movement behaviour promotion, thereby lessening feelings of guilt among adults accessing care. In the abovementioned systematic review [58], researchers, policy-makers, and medical providers felt that PA and SB guidelines could instill guilt among those who do not meet the guideline recommendations. However, they also suggested that defining and providing examples of forms of PA and SB could mitigate confusion and better assist adults in meeting the recommendations [58]. Such definitions and examples could be well-suited to the User Guide or another practical resource to improve understandability of the Tool.

Wanting to use the Tool and User Guide in practice may signify PCPs’ legitimizing of the Tool and User Guide as innovations that ought to be enacted [51]. PCPs suggested how to make the Tool visible to adults accessing care, such as posting it in waiting rooms, which represents PCPs’ acceptance of the Tool [51]. According to NPT, the drive to use the Tool increase its visibility exemplifies PCPs’ cognitive participation to promote embedding of the Tool in future practice [25]. Conversely, PCPs’ barriers related to a lack of future uptake of the Tool and User Guide (another form of cognitive participation). For instance, PCPs underlined that some prompts and ordering of sections felt unnatural and some instructions were unclear. Suggestions to add supporting resources (e.g., a preamble and handout), develop the Tool in an EMR format, and provide more information on the benefits of engaging in the 24HMG behaviours in the Tool and User Guide were made. Fulfilling these suggestions could enhance PCPs’ activation with the Tool and User Guide by improving their usability. Overall, barriers pertained to a potential for low motivation to use the Tool and User Guide unless certain modifications were made. However, research has indicated that implementation barriers may not necessarily prevent routine embedding of an innovation in practice, so long as support exists for coherence, cognitive participation, and collective action [60].

Through the NPT lens of collective action, we can foresee how PCPs could work together in the future to embed the Tool and User Guide within their settings. One aspect of collective action is possessing a workable skill-set to enact an innovation, such as sufficient knowledge or awareness of an innovation [51]. In our sample, PCPs described a need for increased knowledge of how referral would unfold in a discussion about movement behaviours, education about the evidence behind the 24HMG, and even training on the Tool and User Guide, to improve their skill-set. Nevertheless, some PCPs possessed skill-sets relevant to the Tool or the 24HMG, such as a kinesiology degree, which facilitated its use.

### Processes of using the Tool and User Guide

In designing the Tool and User Guide, the intention was for it to guide conversations on movement behaviours that were person-centered and aimed to benefit adults accessing care with or without a health condition(s). Various approaches were taken by PCPs to this end, such as agreeing on relevant and feasible goals [61, 62] or using the scaling questions for readiness to elicit change talk [32, 63]. Additionally, many PCPs stated that aspects of the Tool and User Guide aligned with how they would approach similar conversations in their practice, including the collaborative spin. These examples demonstrate how PCPs both understood what the Tool and User Guide asked of them (individual specification) and valued what the Tool and User Guide offered (internalization), illustrating coherence with the Tool and User Guide [51]. Nevertheless, some PCPs did not grasp the Tool and User Guide’s instructions and took their own approach, which was most evident in PCPs who did not use the open-ended prompts in ASSESS or the scaling question for readiness. As suggested by NPT, this absence of individual specification, whereby PCPs did not understand aspects of the Tool and User Guide’s procedure, showcased the need to improve PCPs’ coherence [51]; clarifying and simplifying instructions within the Tool and User Guide may enhance PCPs’ understanding.

Lastly, PCPs interpretation of how to use the Tool and User Guide varied from wanting to use them only with certain adults (i.e., those who are motivated, those who could benefit the most from lifestyle changes) to wanting to use only certain sections (e.g., ASSESS but not COUNSEL). Per NPT, viewing the ‘work’ of using the Tool and User Guide as feasible for some clinical scenarios but not others constitutes both the presence and lack of interactional workability (a component of collective action) of the Tool and User Guide [25, 51]. In sum, how PCPs operationalize the Tool and User Guide is central to promoting adoption in practice [25, 53].

### Development of the Whole Day Matters Toolkit

With limited space in the Tool and User Guide, we were unable to incorporate all PCPs’ suggestions in our revisions. For instance, given the variability of primary care nationally [64], we were unable to suggest local services for referral as some PCPs desired. However, most suggestions were incorporated into our modifications to enhance usability, acceptability, and future implementation. Given the coherence expressed by PCPs in this study, the majority of the content, structure, and appearance of the Tool and User Guide remained unchanged. Specifically, the spirit of motivational interviewing, the modified 5 A’s framework, 24HMG colours, asking for permission to discuss movement behaviours, the readiness scale, and tips for achieving movement behaviours were retained as important elements to the Tool and User Guide. Further, explaining the relevance of the 24HMG to the adult accessing care was identified as beneficial; thus, the instructions in the ASK and ADVISE sections of the Tool and User Guide were altered to signal PCPs to connect the 24HMG and target behaviour with the adult’s health condition(s) or reason for encounter [65]. To target *in*coherence, we revised language for a lay audience, traded the checkboxes for images in ASSESS, and removed the goal-setting table in the Tool. The term “movement behaviours”, however, was retained in keeping consistent with the 24HMG and directing future work to promote awareness, advocacy, and adoption of terminology related to the 24HMG among PCPs and the general population. Additionally, we reduced text by summarizing and increased white space wherever possible. Finally, in the User Guide, we clarified how to use the readiness scale and removed part of the motivational interviewing prompts to create space for adding definitions of PA intensity and additional benefits to help PCPs discuss the 24HMG recommendations [58].

**Fig. 2:**
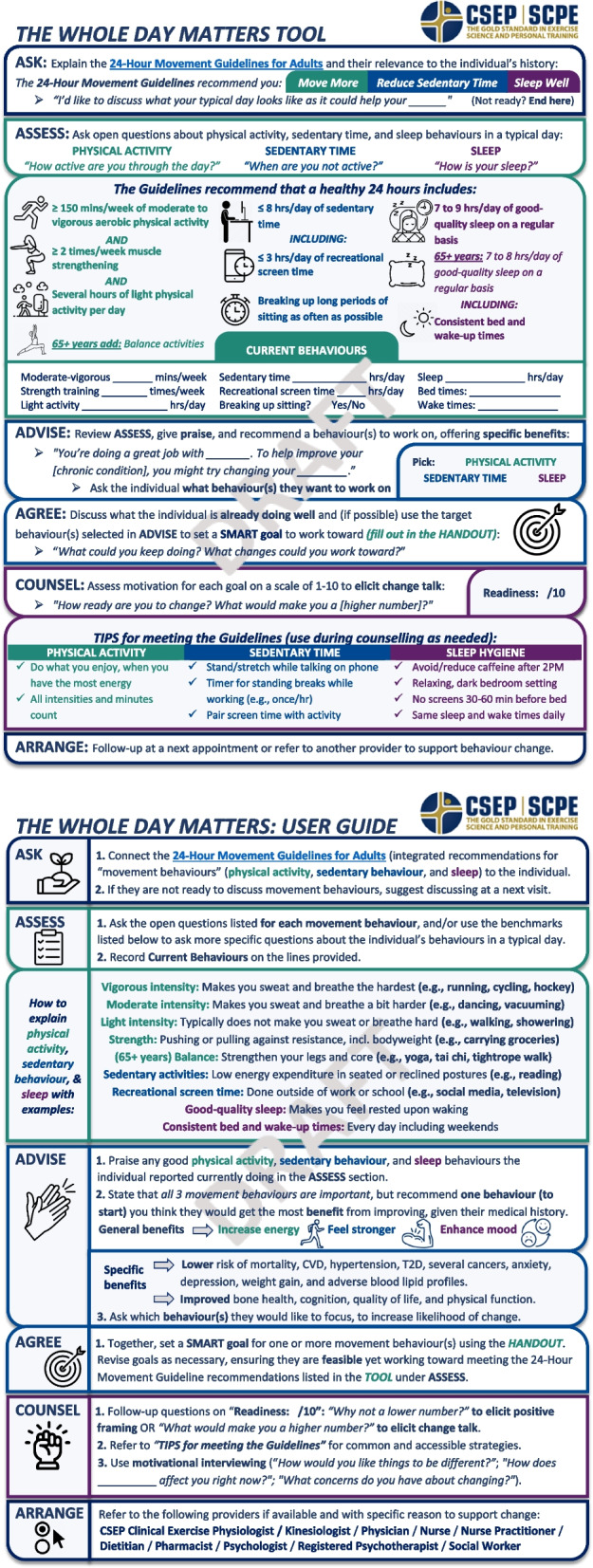
The Whole Day Matters Tool and User Guide post think-aloud

Cognitive participation was not fully supported as PCPs felt the prompts and ordering of sections within the Tool and User Guide were unnatural. Thus, prompts were simplified throughout and the order and titles of Tool sections were restructured as ASK > ASSESS > ADVISE (previously AGREE) > AGREE (previously PRESCRIBE) > COUNSEL > ARRANGE (previously REFER); the User Guide sections were reorganized in tandem. The lack of collective action, seen in PCPs’ low knowledge and awareness of the 24HMG and referral processes, informed additional changes to the Tool and User Guide: links to the 24HMG website were inserted in the ASK section of the Tool to promote self-education about 24HMG evidence. Moreover, PCPs reported confusion about whether the Tool could be used in sections rather than in its entirety and whether one or multiple movement behaviours could be discussed in a single appointment. Accordingly, we added a more formal description of the Tool and User Guide (i.e., a preamble), which we elaborate on below. See Fig. 2 for the revised Tool and User Guide.

**Fig. 3:**
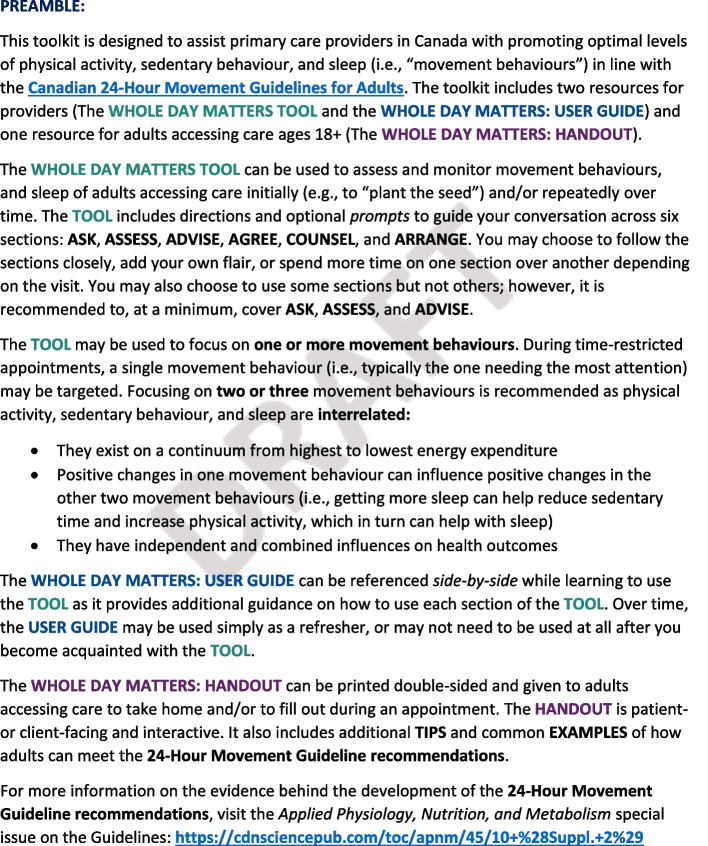
The Whole Day Matters Preamble

**Fig. 4:**
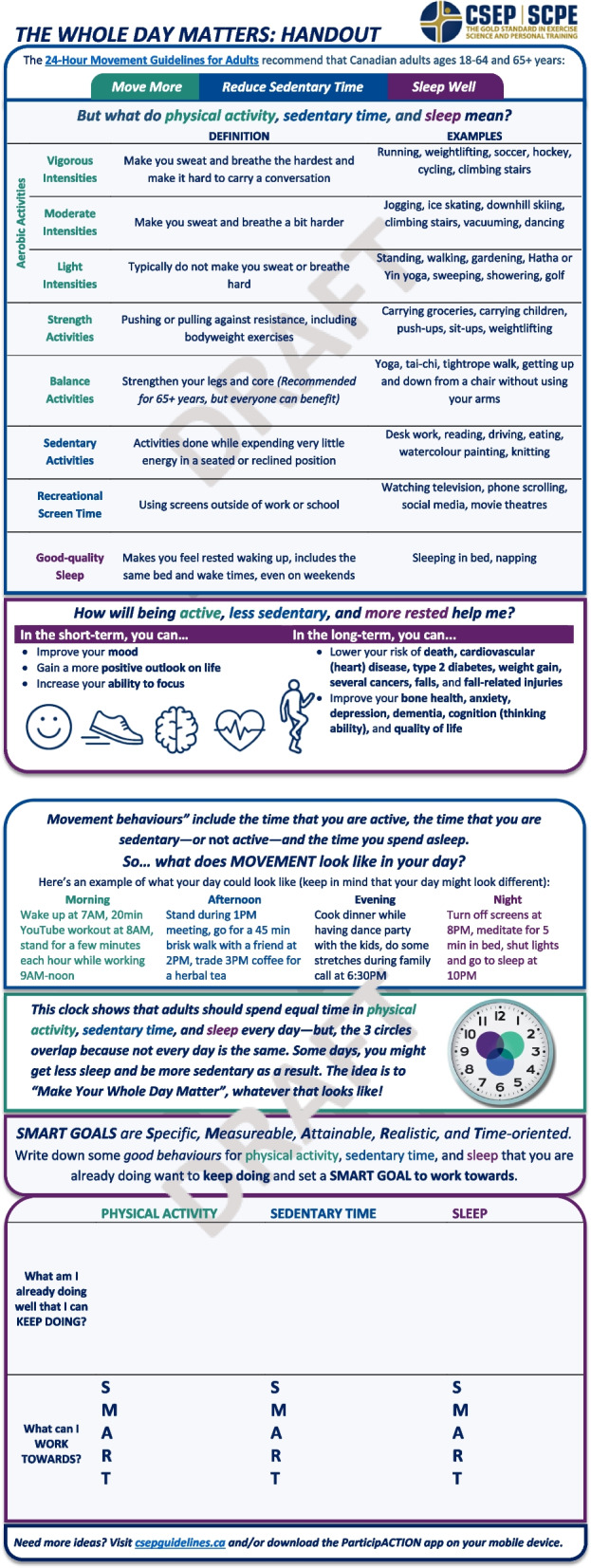
The Whole Day Matters Handout

Finally, in response to PCPs’ recommendations to introduce the Tool and User Guide more formally and enhance person-centeredness, we developed two additional resources: (i) a Preamble (Fig. 3) and (ii) a Handout (Fig. 4). Together, these four resources are titled “The Whole Day Matters Toolkit”. The Handout is a two-page worksheet for adults accessing care including palatable movement behaviour definitions and examples, a list of short- and long-term benefits to meeting the 24HMG, and a fillable SMART (Specific, Measureable, Attainable, Realistic, Time-oriented [66]) goal box that is referenced in the revised Tool. The Preamble is a one-page introduction to the 24HMG and Toolkit, including a link to the 24HMG website and descriptions of the purpose and intended users of the Tool, User Guide, and Handout. Moving forward, we envision the Handout as extending the person-centeredness of the Tool, and the Preamble as clarifying that the Tool bears utility whether used in part or in full and whether one or more movement behaviours are discussed.

### Strengths and limitations

A key asset to this study was its iKT approach involving a working group that contributed to study design, protocol development, and tool revisions. Drawing from their diverse perspectives in medicine, exercise psychology, health promotion, communications, and KT allowed for balanced discussion at all research stages, which should enhance the relevancy of our findings to the local context [28]. Observing the usability of the Tool and User Guide in think-aloud and near-live procedures was another strength as few tools covering one or two movement behaviours have undergone usability testing [16]. Further, we are unaware of any other studies using think-aloud and near-live methods sequentially and within the same sample. Combining the two methods was beneficial to our purposes as it provided access to PCPs’ perceptions of usability broadly, via both their working memory and simulations that mimicked primary care practice. More specifically, the combined methods allowed us to identify and follow-up on instances where PCPs, during the near-live task, may have acted in opposition to what they said during the think-aloud task. Moreover, we were able to access PCPs’ cognition on superficial and deep usability problems and their insight in both their first-time use of, and after becoming familiar with, the Tool and User Guide. Indeed, PCPs stressed the need to familiarize oneself with the Tool and User Guide prior to use in practice. Moreover, we followed Tracy’s [67] criteria for high quality in our qualitative design, whereby the theoretical application, data collection, and analysis were rigorous, the methods and procedures meaningfully cohered with our study goals, and the critical friend enhanced the credibility of our findings. Finally, the topic was worthy and timely as the Tool and User Guide fill a gap in PCPs practice to guide integrated discussions on the 24HMG since their release in 2020 [67].

There were several limitations to this study. Despite aiming to recruit an equal number across five categories of PCPs (i.e., family medicine physicians/residents, nurses/nurse practitioners, dietitians, pharmacists, psychologists/registered psychotherapists/social workers), most participants were family medicine residents. Furthermore, some professions were excluded, such as qualified exercise professionals and physiotherapists, who may be integrated within primary care settings in some contexts. Therefore, findings may not apply to all PCP populations, which could influence Toolkit implementation. Moreover, PCPs were recruited from Kingston, Ontario, which could limit the resonance of our findings [67]. Finally, content analysis was performed on manifest data only; therefore, insights from any latent data were possibly missed.

### Implications

Since study culmination, the working group has sought expert consensus on a final version of the Toolkit from a more representative sample of PCPs in a modified Delphi study (publication forthcoming), who encompassed a wider distribution of PCP populations and geographic areas. The final Toolkit was launched on September 21^st^, 2022 [68]. Several dissemination reach metrics are currently being evaluated and will be reported in the forthcoming final Toolkit publication. The Toolkit has also been implemented in the local medical curriculum for health promotion learning purposes. This study has various implications for PCPs and adults accessing care, including helping PCPs discuss the 24HMG more frequently and with greater knowledge, skill, and confidence, which may then improve adults’ movement behaviours. Additionally, other researchers may wish to adopt a similar methodological approach in developed or refining other health care tools. However, Toolkit effectiveness has yet to be assessed. Thus, future research evaluating the Toolkit’s efficacy to improve 24HMG discussions in primary care, learning outcomes in medical education, and behavioural and health outcomes are worthy next steps.

## Conclusion

The Whole Day Matters Tool and User Guide are complementary resources that guide PCPs through discussions on the 24HMG. Positive and negative aspects were identified, pertaining to the content, function, flow, and appearance of the Tool and User Guide. Barriers, facilitators, and varying approaches to future use of the Tool and User Guide were also highlighted. Based on PCPs’ perceptions of usability, acceptability, and future implementation, adaptations were made and a Preamble and Handout were developed to enhance the relevancy of the Tool and User Guide to PCPs. The “Whole Day Matters Toolkit” for primary care has undergone further revision in the abovementioned consensus building process and has been incorporated into the local medical curriculum. The Whole Day Matters Toolkit is now available to PCPs looking to incorporate or improve movement behaviour discussions in their practice, or medical educators looking to teach about movement behaviour promotion, to optimize physical and mental health outcomes of adults accessing care.

## Supplementary Information

**Additional file 1. **Protocol for think-aloud procedure and near-live scenarios.

**Additional file 2. **Mock service-user profiles for near-live scenarios.

**Additional file 3. **Interview guide including Normalization Process Theory.

**Additional file 4: S-table 1. **Standards of Reporting in Qualitative Research (SRQR) Checklist.

## Data Availability

The data used and analysed during the current study are available from the corresponding author on reasonable request.

## Abbreviations

24HMG: 24-Hour Movement Guidelines for Adults
iKT: Integrated knowledge translation
KT: Knowledge translation
NPT: Normalization Process Theory
PA: Physical activity
PCPs: Primary care providers
SB: Sedentary behaviour
SMART goal: Specific, Measureable, Attainable, Realistic, Time-oriented
