# Supplementary material for: Developing and testing the usability, acceptability, and future implementation of the Whole Day Matters Tool and User Guide for primary care providers using think-aloud, near-live, and interview procedures
Source: BMC Med Inform Decis Mak. 2023 Apr 6;23:57. doi: 10.1186/s12911-023-02147-x (PMC10080928; doi:10.1186/s12911-023-02147-x)
Supplement: Supplementary file 1 — Additional file 1. Protocol for think-aloud procedure and near-live scenarios. [file 12911_2023_2147_MOESM1_ESM.docx]

**Additional File 1.** Protocol for think-aloud procedure and near-live scenarios.

**Warm-Up Task**

Thank you for volunteering to take part in this study related to the development of a 24-Hour Movement Guideline discussion tool for health care providers.

The first part of today’s session is called a “think aloud” task. The purpose of a think aloud is to obtain a record of your thoughts as you complete a task. As you perform the task, please think out loud continuously, verbalizing any and all thoughts that come to mind. I may interrupt you to remind you of the instructions, but there will otherwise be minimal communication from me during the task.

We will begin with a warm-up task to practice thinking out loud. It is completely unrelated to the study and will take approximately 2 minutes.

Do you have any questions before we begin?

**Prompt:** Imagine you are about to write and send an email. Please say everything that would go through your mind during that process.

*Note: The observer may provide some feedback on the participant’s performance after the warm-up exercise.*

**Think-Aloud Task**

Thank you for completing the warm-up task. The next part of the think-aloud is related to the 24-Hour Movement Guideline discussion tool. It will take approximately 5 minutes.

Our objective is to understand how the tool could be used in clinical practice. As you interpret the tool, please consider factors that relate to its utility. As a reminder, we ask that you think out loud continuously during the exercise. I will start by sharing the tool on my screen, giving you mouse-control, and reading you a prompt.

Do you have any questions before we begin?

**Prompt**: I am screen-sharing a 1-page discussion tool and a 1-page user guide; the user guide has explanations and instructions for how to use the discussion tool, and example prompts for what you could say in a given section. Imagine you are using the discussion tool provided on page 1 to guide a conversation on any or all movement behaviours with a 45-year-old male client who has fatigue or mood changes and no other acute health concerns. Please say everything that goes through your mind as you interpret the tool and let me know when you are finished. You may begin.

**Near-Live Task**

Thank you for completing the think-aloud task. For this next part, you will continue to use the 24-Hour Movement Guideline discussion tool, but will be interacting with a mock client. It will take approximately 5 minutes.

Our objective is to understand how the tool might be used during a conversation in a simulated setting. Please try your best to use the tool to guide your conversation with the mock client as if you were talking with a real client. In this task, we ask that you **do not** think out loud, but rather talk how you normally would during a real encounter. I will share the tool on my screen again and will give you mouse control.

Do you have any questions before you begin?

**Physical activity prompt**: You are seeing 25-year-old female client who has fatigue and type 1 diabetes. Their job involves sitting at a desk most of the day, and at home they are typically quite busy taking care of their child. The history you take does not suggest the individual is living with any chronic conditions **other than diabetes with an A1c at target**, does not meet the criteria for a depressive or anxiety disorder, and you have no acute concerns about their health, other than an issue that could be addressed with the 24-Hour Movement Guidelines. Given this, you would like to focus the discussion on one movement behaviour.

Please use the discussion tool to guide your conversation with the mock client and let me know when you are finished. You may begin.

**Sedentary behaviour prompt**: You are seeing 25-year-old female client who has fatigue. Their job involves sitting at a desk most of the day, and at home they are typically quite busy taking care of their child. The history you take does not suggest the individual is living with any chronic conditions, does not meet the criteria for a depressive or anxiety disorder, and you have no acute concerns about their health, other than an issue that could be addressed with the 24-Hour Movement Guidelines. Given this, you would like to focus the discussion on one movement behaviour.

Please use the discussion tool to guide your conversation with the mock client and let me know when you are finished. You may begin.

**Sleep prompt**: You are seeing 25-year-old female client who has mood changes. Their job involves sitting at a desk most of the day, and at home they are typically quite busy taking care of their child. The history you take does not suggest the individual is living with any chronic conditions, does not meet the criteria for a depressive or anxiety disorder, and you have no acute concerns about their health, other than an issue that could be addressed with the 24-Hour Movement Guidelines. Given this, you would like to focus the discussion on one movement behaviour.

Please use the discussion tool to guide your conversation with the mock client and let me know when you are finished. You may begin.
