# Supplementary material for: Developing and testing the usability, acceptability, and future implementation of the Whole Day Matters Tool and User Guide for primary care providers using think-aloud, near-live, and interview procedures
Source: BMC Med Inform Decis Mak. 2023 Apr 6;23:57. doi: 10.1186/s12911-023-02147-x (PMC10080928; doi:10.1186/s12911-023-02147-x)
Supplement: Supplementary file 2 — Additional file 2. Mock service-user profiles for near-live scenarios. [file 12911_2023_2147_MOESM2_ESM.docx]

**Additional File 2.** Mock service-user profiles for near-live scenarios.

**Near-live**

**2 groups:**

1. Intenders (low risk)—**will** agree to setting a goal and to any follow-up plans set by the provider participant
2. Non-intenders (high risk)—**will not** agree to setting a goal, but **will** agree to follow-up plans set by the provider participant

**PHYSICAL ACTIVITY**

The mock client will express that they have felt very fatigued recently (“I’ve had diabetes for a while, but I’ve been feeling more tired than usual lately”). When asked by the participant to have a discussion about Canada’s 24-Hour Movement Guidelines, they will agree. When asked about their current movement behaviours, they will state that (1) they “don’t really work out or anything but I move around with my kid on the weekends”, **and** (2) “I sit at my desk all day and watch tv at night”, **and** (3) “I usually get 7 hours of sleep, which is enough for me to feel rested”.

***(Intender)*** The mock client will agree to focus on one of their current movement behaviours. They will express an interest in physical activity (“I know I *should* be doing physical activity, but I feel like I never have the time or energy for it”) and will agree to negotiate a realistic goal with the participant. When asked about barriers to physical activity, the mock patient will raise the issue of their busy schedule (“I just don't know how I’d ever fit it in”). The mock client will agree to any follow-up plans proposed by the participant.

***(Non-intender)*** The mock client will agree to focus on one of their current movement behaviours. They will agree on physical activity, but will express they do not intend to change anytime soon (“I guess I could be more active, do more exercise. I know I *should* be doing physical activity, I just never have the energy for it and I like watching my shows”). The patient will not want to negotiate a goal with the participant. When asked about barriers to physical activity, the mock patient will raise the issue of their busy schedule (“I just don't know how I’d ever fit it in”). The mock client will agree to any follow-up plans proposed by the participant.

**SEDENTARY BEHAVIOUR**

The mock client will express that they have felt very fatigued recently (“I feel so tired all the time, like I’m dragging my feet around”). When asked by the participant to have a discussion about Canada’s 24-Hour Movement Guidelines, they will agree. When asked about their current movement behaviours, they will state that (1) they “go for walks occasionally”, **and** (2) “I sit at my desk all day and just feel like relaxing when I get home”, **and** (3) “I usually get 7 hours of sleep, which is enough for me to feel rested”.

***(Intender)*** The mock client will agree to focus on one of their current movement behaviours. They will express that they want to focus on sedentary behaviour (“I know I *should* get moving more, but it’s hard to fit in with my schedule right now”) and will agree to negotiate a realistic goal with the participant. When asked about barriers to reducing sedentary behaviour, the mock patient will talk about their work situation (“I just don't know how to incorporate it into my day at work”). The mock client will agree to any follow-up plans proposed by the participant.

***(Non-intender)*** The mock client will agree to focus on one of their current movement behaviours. They will agree on sedentary behaviour, but will express they do not intend to change anytime soon (“I guess I could work on how much I’m sitting. I know I *should* get moving more, but I just don’t think I can do that on my schedule right now”). The patient will not want to negotiate a goal with the participant. When asked about barriers to reducing sedentary behaviour, the mock patient will talk about their work situation (“I just don't know how to incorporate it into my day at work”). The mock client will agree to any follow-up plans proposed by the participant.

**SLEEP**

The mock client will express that they have noticed a change in their mood recently (“I don’t know why, but I feel irritable most days”). When asked by the participant to have a discussion about Canada’s 24-Hour Movement Guidelines, they will agree. When asked about their current movement behaviours, they will state that 1) “I go for walks pretty regularly”, **and** (2) “I sit at my desk all day, but then I’m up and moving with my kid during the evenings and weekends”, **and** (3) “I usually get 5 hours of sleep and try to catch up on the weekends if I can.”

***(Intender)*** The mock client will agree to focus on one of their current movement behaviours. They will express an interest in focusing on sleep (“I’d like to focus on getting better sleep. I know I should sleep more, there just aren’t enough hours in the day”) and will negotiate a realistic goal with the participant. When asked about barriers to sleep, the mock patient will raise the issue of their busy schedule (“I’m pretty busy between work and my kid, and late at night is when I go on my computer to catch up on things”). The mock client will agree to any follow-up plans proposed by the participant.

***(Non-intender)*** The mock client will agree to focus on one of their current movement behaviours. They will agree on sleep, but will express they do not intend to change anytime soon (“My sleep isn’t great, but I can manage. I know I should sleep more, but it’s just not something I feel like I can change”). The patient will not want to negotiate a goal with the participant. When asked about barriers to sleep, the mock patient will raise the issue of their busy schedule (“I’m pretty busy between work and my kid, and late at night is when I go on my computer to catch up on things”). The mock client will agree to any follow-up plans proposed by the participant.
