# Supplementary material for: Developing and testing the usability, acceptability, and future implementation of the Whole Day Matters Tool and User Guide for primary care providers using think-aloud, near-live, and interview procedures
Source: BMC Med Inform Decis Mak. 2023 Apr 6;23:57. doi: 10.1186/s12911-023-02147-x (PMC10080928; doi:10.1186/s12911-023-02147-x)
Supplement: Supplementary file 3 — Additional file 3. Interview guide including Normalization Process Theory. [file 12911_2023_2147_MOESM3_ESM.docx]

**Additional File 3.** Interview guide including Normalization Process Theory.

**Debriefing Interview Guide**

**Opening Statement:** Thank you for volunteering to take part in this study related to the development of a 24-Hour Movement Guideline discussion tool for health care providers. Our objective is to better understand your perceptions of the tool’s usability and how it performed when tested in a simulated clinical encounter. There are no right or wrong answers and you can refuse to answer any question you don’t wish to answer. This discussion should last approximately 10-15 minutes. Do you have any questions before we begin?

*Reminder to the interviewer: summarize the participant’s comments after each section.*

**Reflections on the Tool from the Think-Aloud**

- What are your thoughts on the usability of the tool based on your experience during the first (think-aloud) task?
- Can you explain the difficulties you had with [refer to section of the tool based on field notes]? (Alternatively, “Can you explain any difficulties you had with the tool?”)
- Can you elaborate on what you were thinking when [refer to moment during the think aloud based on field notes]?

*The questions above may be repeated for more than one section of the tool.*

**Reflections on the Tool from the Near-Live Simulated Patient Encounter**

- How was your experience using the tool with the mock patient?
- Can you explain the difficulties you had with [refer to moment during the simulated encounter]? (Alternatively, “Can you explain any difficulties you had using the tool to guide your discussion?”)
- Can you elaborate on what you were thinking when [refer to moment during the simulated patient encounter based on field notes]?

*Questions may be repeated in reference to more than one instance in the simulated encounter.*

- Can you envision how you might use the tool with real clients in your practice?

**Questions on Additional Resources**

- (NPT “coherence”) What did you find most meaningful or useful about the tool?
  - (NPT) What did you find least meaningful or useful?
- (NPT “cognitive participation”) What additional information or resources might you need to commit to using the tool in your practice?
  - (NPT “collective action”) What factors might promote or inhibit your use of the tool in your practice?
- We have considered the possibility of creating an educational resource to accompany the tool that includes background information about what the 24-Hour Movement Guidelines are and the evidence supporting the Guideline recommendations.
  - Do you think this would help you or other health care providers use the tool?
  - Do you have any other thoughts that you want to share?

**Concluding Remarks:**

This brings us to the end of our meeting. Is there anything else you would like to add?

Again, we would like to thank you for your participation in this study. You have provided valuable information, which may lead to improvements of the 24-Hour Movement Guideline discussion tool. I will follow-up in the coming days by email to give you your $75 compensation, where I will ask which company or charity you would like your e-gift card to go towards. Just to confirm, your email address is [confirm participant email address]?

Thank you and take care (end of interview).
